# Microbiome and One Health: Potential of Novel Metabolites from the Gut Microbiome of Unique Species for Human Health

**DOI:** 10.3390/microorganisms11020481

**Published:** 2023-02-15

**Authors:** Ruqaiyyah Siddiqui, Naveed Ahmed Khan

**Affiliations:** 1 College of Arts and Sciences, American University of Sharjah, University City, Sharjah 26666, United Arab Emirates; 2 Department of Medical Biology, Faculty of Medicine, Istinye University, Istanbul 34010, Turkey; 3 College of Medicine, University of Sharjah, University City, Sharjah 27272, United Arab Emirates

For thousands of years, the notion that human health and performance are concomitant with the health and diversity of the microbiome has been deliberated upon [1]. Research on the microbiome has revealed that the health and diversity of the gut microbiome and overall health of the host are correlated. The microbiome refers to the trillions of microorganisms inhabiting their hosts and contains up to 80% of the hosts’ immune system. It is the gut microbial metabolites which link the microbiota and the immune system and modulate various aspects of immune cell function through a plethora of mechanisms [2,3]. Gut microbial dysbiosis has been associated with various pathologies such as cancer, infectious diseases, ageing, obesity, and more recently the gut microbial changes pertaining to female health issues are beginning to come to light [4,5,6,7]. Given that there is a dearth of studies on females, future studies to understand gut microbial changes during menopause, pregnancy, menstruation, osteoarthritis, and cancer are warranted. 

Another fascinating area of research is the effect of the microbiome in microgravity environments. Since 1961, hundreds of astronauts have ventured into space, and there is much interest in future prolonged space missions. This is evidenced by the recent report by 20 national space agencies denoted as the Global Exploration Roadmap [8], which details an approach to upsurge in activity in the space, as well as the aim of colonizing Mars by 2050, in addition to undertaking regular spaceflights by visitors [9]. This is despite the fact that space flights can instigate a plethora of stressors leading to negative health effects associated with exposure to radiation, microgravity, and the imbalance in physiological responses of the human body, along with dysbiosis of the gut microbiome, thus affecting overall astronaut health and performance [10]. In particular, research over the past few decades has emphasized the increased exposure to radiation and subsequent immune dysregulation that has been linked clearly with the space missions [11,12,13,14]. As immunity is connected with all other physiological systems, immune system dysregulation can lead to associated pathologies. 

*Homo sapiens* are merely one species residing on this planet, amongst millions, and several studies have focused on the effect of the gut microbiome in humans. Studying the gut microbiome of animals has received much less attention. Nonetheless, with the awareness of the concept of one health, which refers to the connection of human, animal, plant, and environmental health, it is logical to investigate the gut microbiome of unique and interesting animals such as long-lived animals including reptiles who have evolved, adapted and survived successfully over millions of years, dwelling in often harsh environments containing heavy metals and coming across radiation [15]. Other species such as cockroaches are of interest, given their status as one of the most successful and diverse insects. Their gut microbial metabolites have shown anti-bacterial effects thus offer a novel arena for biodiscoveries for the benefit of human health and may be potentially used as probiotics and/or antibiotics [16]. Recently, a plethora of molecules depicting potent inhibitory properties were identified from crocodiles, namely; l,l-Cyclo(leucylprolyl). l,l-cyclo(leucylprolyl), a flavonoid that possesses anticancer effects. Other molecules comprised lactic acid, f-Honaucin A, l,l-Cyclo(leucylprolyl), and 3-hydroxy-decanoic acid, however, the properties and activity of the majority of molecules remain unidentified and are the subject of prospective experimentation [17]. Another study revealed four highly active peptides identified from crocodile meat, that possessed free radical scavenging activities [18]. Moreover, another study on the water monitor lizard revealed several metabolites from the gut bacteria, belonging to the class of flavonoids, terpenoids, alkaloids, polyhydroxy alkaloids, polyacetylenes, bisphenols, amides, oxylipin and pyrazine derivatives with broad-spectrum antimicrobial, anti-oxidant, anti-tumor, anti-inflammatory, and analgesic characteristics [19]. Hence, there is a need to identify molecules/mechanisms of translational value from such unique species and extrapolate metabolites/molecules to augment human health and performance, ageing, longevity and female health, as well as strengthen the immune system during space flights, to improve astronaut health. Herein we deliberate some of the stressors that may arise in gut microbial dysbiosis, due to these issues and suggest that we should look to “hardy and resilient” species such as cockroaches and crocodiles and analyze their protective/defense mechanisms to improve human health and human performance, given the concept of one health. We propose that the development of pre/pro/postbiotics from these remarkable species can be successfully applied to humans for the prevention or restoration of gut dysbiosis and thus will be beneficial for overall health. 

## What Can We Learn from Hardy Species?

Although radiation is not limited to space, human exposure to radiation is far greater in space than on Earth. For example, humans would receive approximately 400–900 millisievert annually in inter-planetary space compared with 2.4 millisievert on the Earth. A recent study showed that mice are twice as likely of developing cancer due to radiation exposure following a Mars mission [20]. Even with exposure of 2.4 millisievert on Earth, the incidence rate of cancer in humans can be up to 50% (albeit it is attributed to several factors including diet, lifestyle, environmental conditions, etc.), despite the advent of radiation protection strategies such as creams, glass, clothing, etc. 

It is fascinating to observe that species such as cockroaches, crocodiles, tardigrades can tolerate elevated radiation, dwell in unhygienic conditions, feed on germ-infested diet, and frequent heavy metals exposure. Interestingly, they have seldom described developing diseases such as cancer [21]. For example, tardigrades are now considered one of the most resilient species on Earth that can endure high levels of radiation, dehydration, being frozen for over a year and temperatures ranging from zero to 100 °C, tolerate six times more pressures than at the bottom of the ocean. Their ability to shield DNA from radiation damage is remarkable. Although the precise molecular mechanisms are unclear, in part, this is due to a protein known as Dsup (damage suppressor) that can (i) physically insulate DNA without affecting its function, and (ii) clear reactive oxygen species, leading to enhanced tolerance to radiation [22]. The findings that overexpression of Dsup in human cells prevented them against radiation damage are promising and offer tremendous opportunities. Cells expressing Dsup exhibited up to 50% less damage to the DNA by X-rays, in comparison to the controls. In addition, several protective genes/enzymes that neutralize reactive oxygen species and/or repair DNA such as *MRE11* were observed. These findings offer a promising avenue in that the radiation tolerance of humans during space missions can be enhanced through transfer of *Dsup* using genetic engineering. Similarly, cockroaches are one of the few species that can survive high levels of radiation, i.e., 10,000 rads that is atomic bomb level. Cockroaches were found to thrive 1000 feet away from where the Hiroshima atom bomb was dropped. Of note, hundreds of compounds were found from cockroach lysates [23] and few of the compounds were identified and found to contain molecules with isoquinoline group, chromene derivatives, thiazine groups, imidazoles, pyrrole-containing analogs, sulfonamides, furanones, and flavanones, displaying broad-spectrum antimicrobial properties and anti-inflammatory, anti-tumour, and analgesic properties [23]. This was followed up by a subsequent study which examined gut bacterial metabolites from cockroaches [24]. Several molecules were elucidated such as syringetin 3-galactoside, a flavonol and actinamine, cucurbitacin Q, that had known biological activities [24]. Several molecules with no known biological activities were also identified, comprising L-Hexahydro-3-imino-1,2,4-oxadiaze-pine-3-carboxylic acid, 5, 8-Dihydroxy-6,7,4’-trimethoxy-flavone 8-glucoside, Tyr Leu Arg, 5-Oxododecanoic acid [24]. Another study revealed a large repertoire of surfactin and iturin A (lipopeptides) molecules from the gut microbiome of rats [25]. To our knowledge, this is the first report of isolation of lipopeptides from bacteria isolated from the rat gut. Other species, for instance crocodiles are always exposed to radiation (sun), heavy metals, and other carcinogenic materials. It is suggested that these species have evolved strategies to counter cancer development or contracting diseases. Moreover, *H. sapiens* are a moderately recent addition to this planet, while other species have been inhabiting this planet for millions of years. How species such as the crocodile can persist for almost a century and endure carcinogenic conditions which are damaging to humans is a puzzle. These species have most likely evolved with mechanisms to defend themselves against cancer development. There are two logical explanations for these findings: (i) such remarkable animals have developed a robust immunity, and/or, (ii) microbial flora of the mouth/gastrointestinal tract of the aforementioned species yields novel biomaterials. Based on these findings, it is compelling to analyze mechanisms or sequester their bioactive molecules for human health and wellbeing. 

Taken together, a plethora of studies indicate the pivotal role of the microbiome in controlling the host’s health and contributes significantly to modulating human health and performance. Thus, the development of strategies to restore gut microbial diversity and maintain the precise ratios of bacterial species within the gut for optimal health are required and should be investigated in future space missions as well as in analog missions, or ground based in vivo and in vitro models of microgravity/stress/ageing or various models of disease. Increased understanding of the novel molecules produced by fascinating species such as cockroaches and crocodiles and their precise and detailed effects on ageing, longevity, cancer, cellular senescence or overall human health are warranted [15]. Recent studies have depicted the potent anticancer and antibacterial nature of molecules from these species [26]. In particular, LC-MS will be a valuable approach to carryout metabolomics and proteomics studies to identify potential bioactive molecules. This combined with nuclear magnetic resonance (NMR) spectroscopy can provide insight into their molecular identity. These molecules can then potentially be developed as prebiotics and/or postbiotics to protect humans against stressors of the space environment and tested in vivo microgravity/stress models, such as the unloading model [27]. Such novel studies will pave the way in our search for the development of novel pre/pro/postbiotics and/or other therapies. This is strengthened by the concept of one health which denotes that the health of humans, plants and animals and the overall environment are interconnected.
